# Direct sequencing and expression analysis of a large number of miRNAs in *Aedes aegypti *and a multi-species survey of novel mosquito miRNAs

**DOI:** 10.1186/1471-2164-10-581

**Published:** 2009-12-04

**Authors:** Song Li, Edward A Mead, Shaohui Liang, Zhijian Tu

**Affiliations:** 1 Department of Biochemistry, Virginia Polytechnic Institute and State University, Blacksburg, VA 24061, USA; 2 Department of Parasitology, Wenzhou Medical College, Wenzhou, Zhejiang Province, PR China

## Abstract

**Background:**

MicroRNAs (miRNAs) are a novel class of gene regulators whose biogenesis involves hairpin structures called precursor miRNAs, or pre-miRNAs. A pre-miRNA is processed to make a miRNA:miRNA* duplex, which is then separated to generate a mature miRNA and a miRNA*. The mature miRNAs play key regulatory roles during embryonic development as well as other cellular processes. They are also implicated in control of viral infection as well as innate immunity. Direct experimental evidence for mosquito miRNAs has been recently reported in anopheline mosquitoes based on small-scale cloning efforts.

**Results:**

We obtained approximately 130, 000 small RNA sequences from the yellow fever mosquito, *Aedes aegypti*, by 454 sequencing of samples that were isolated from mixed-age embryos and midguts from sugar-fed and blood-fed females, respectively. We also performed bioinformatics analysis on the *Ae. aegypti *genome assembly to identify evidence for additional miRNAs. The combination of these approaches uncovered 98 different pre-miRNAs in *Ae. aegypti *which could produce 86 distinct miRNAs. Thirteen miRNAs, including eight novel miRNAs identified in this study, are currently only found in mosquitoes. We also identified five potential revisions to previously annotated miRNAs at the miRNA termini, two cases of highly abundant miRNA* sequences, 14 miRNA clusters, and 17 cases where more than one pre-miRNA hairpin produces the same or highly similar mature miRNAs. A number of miRNAs showed higher levels in midgut from blood-fed female than that from sugar-fed female, which was confirmed by northern blots on two of these miRNAs. Northern blots also revealed several miRNAs that showed stage-specific expression. Detailed expression analysis of eight of the 13 mosquito-specific miRNAs in four divergent mosquito genera identified cases of clearly conserved expression patterns and obvious differences. Four of the 13 miRNAs are specific to certain lineage(s) within mosquitoes.

**Conclusion:**

This study provides the first systematic analysis of miRNAs in *Ae. aegypti *and offers a substantially expanded list of miRNAs for all mosquitoes. New insights were gained on the evolution of conserved and lineage-specific miRNAs in mosquitoes. The expression profiles of a few miRNAs suggest stage-specific functions and functions related to embryonic development or blood feeding. A better understanding of the functions of these miRNAs will offer new insights in mosquito biology and may lead to novel approaches to combat mosquito-borne infectious diseases.

## Background

MicroRNAs (miRNAs) are approximately 22 nucleotide long, non-coding RNAs that regulate the expression of cellular genes by binding to target mRNAs for cleavage or translational repression (reviewed in [1]). Thousands of miRNAs have been reported in animals and plants [2]. Many miRNAs are highly conserved across divergent species while others are specific to a particular evolutionary lineage (miRBase: http://microrna.sanger.ac.uk/). Lineage-specific miRNAs can arise from non-miRNA sequences, which has been observed in *Drosophila*, or by modifications of existing miRNAs [3,4]. MiRNA genes can occur in intergenic regions and within introns [5-8]. For some miRNAs, their biogenesis starts with transcription of miRNA genes, mostly by RNA polymerase II, which produces a primary miRNA (reviewed in [9]). A given primary miRNA can be either monocistronic, containing one mature miRNA, or polycistronic, containing multiple mature miRNAs. In *Drosophila*, the primary miRNA is processed by a Drosha-Pasha complex to yield small stem-loop structures that are approximately 70 nucleotides in length called pre-miRNAs [10]. Following export to the cytosol, the double-stranded pre-miRNA is recognized by Dicer-1, which complexes with Loqs and produces a ~22 bp duplex with 2 nt 3' overhangs [10]. The product is referred to as a miRNA:miRNA* duplex, which is further separated by a helicase and normally the miRNA* strand is rapidly degraded [5,9,11]. During "deep" sequencing studies, approximately 100 fold as many miRNAs as miRNA*s was observed for a given miRNA except in cases where both strands could generate functional miRNAs [7,12,13]. A new category of pre-miRNAs known as mirtrons are derived from introns and undergo Drosha-independent processing [14-16]. Some researchers suggest that the presence of mirtrons indicates that RNA sources may be less relevant for determining which RNAs become miRNAs than their structural characteristics [7]. Mature miRNAs pair with target mRNAs with the assistance of Argonaute to achieve translational repression and/or mRNA degradation (reviewed in [17]).

MicroRNAs play key regulatory roles during embryonic development, stem cell division, cancer development, neurogenesis, heart development, haematopoietic cell differentiation, and cell death (reviewed in [1,18]). They are also implicated in control of viral infection in vertebrates and in one recent report miRNAs were linked to malaria infection in mosquitoes [19-21]. Similarly, miRNAs are also linked to innate immunity (reviewed in [22]). There are reports describing *Anopheles gambiae *miRNAs on the basis of computational prediction or similarity to known miRNAs from other species [23-26]. Direct experimental evidence for mosquito miRNAs has been recently reported only in anopheline mosquitoes using small-scale sequencing [21,27]. There is also a study on genes involved in small RNA pathways in mosquitoes [28].

Using high throughput sequencing and bioinformatics approaches, we performed the first systematic analysis of miRNAs in *Ae. aegypti*. In the current study, we substantially expanded the list of miRNAs for all mosquitoes and uncovered a number of novel and mosquito-specific miRNAs. Northern and small RNA sequencing revealed several miRNAs that may play important roles during embryonic development and during blood feeding. Our analysis also offered insights into the evolution of conserved and lineage-specific miRNAs in mosquitoes.

## Results and discussion

### Discovery of 98 distinct pre-miRNA sequences in *Ae. aegypti*

As shown in Table 1, we have uncovered 98 different pre-miRNAs in *Ae. aegypti *which could produce 86 distinct miRNAs. Some of the 98 pre-miRNA sequences produce identical miRNAs and miRNA*s. Also included in Table 1 are 20 distinct miRNA* sequences that were uncovered by small RNA sequencing. Eighty-nine of the *Ae. aegypti *miRNA and miRNA* sequences showed a perfect match to small RNA sequences from at least one of the three samples (Table 1). There are clear variations of sequence counts among different miRNA species in these samples. However, to gain quantitative insights in the relative abundance of these miRNAs, further investigations are needed using methods such as northern blot, primer extension, and direct sequencing of millions of small RNA reads [11,12,21]. Twenty-nine of the 98 pre-miRNAs do not have small RNA sequences in the embryo and midgut samples. However, these 29 pre-miRNAs all form hairpins and are conserved among *Ae. aegypti*, *Cx. quinquefasciatus*, and *An. gambiae*, the three mosquito species with sequenced genomes.

**Table 1:** Sequence, location, and expression of miRNAs in *Aedes aegypti*.

**Name**^**1, 2**^	**mirBase Name**	**Sequence**^**3**^	**Contig**^**4**^	**Start**^**4**^	**End**^**4**^	**Strand**	**Embryo**^**5**^	**Gut_SF**^**5**^	**Gut_BF**^**5**^
aae-miR-M1-1	aae-miR-2943-1	TTAAGTAGGCACTTGCAGGCAAA	CONTIG_15246	25749	25829	+	28	0	0
aae-miR-M1-2	aae-miR-2943-2	TTAAGTAGGCACTTGCAGGCAAA	CONTIG_15246	25911	25981	+	28	0	0
aae-miR-M2	aae-miR-2942	TATTCGAGACTTCACGAGTTAAT	CONTIG_11944	151669	151750	+	0	0	7
aae-miR-M3	aae-miR-2945	TGACTAGAGGCAGACTCGTTTA	CONTIG_2929	123975	124056	+	33	0	6
aae-miR-M3*	aae-miR-2945*	AGCGGGTCCGTTTCTAGTGTCATG	CONTIG_2929	123975	124056	+	3	0	0
aae-miR-M4a	aae-miR-2944a	GAAGGAACTTCTGCTGTGATCTGA	CONTIG_18252	93868	93929	+	91	2	0
aae-miR-M4a*	aae-miR-2944a*	TATCACAGTAGTTGTACTTTAA	CONTIG_18252	93868	93929	+	3	0	0
aae-miR-M4b	aae-miR-2944b	GAAGGAACTCCCGGTGTGATATA	CONTIG_18252	93731	93792	+	62	0	0
aae-miR-M4b*	aae-miR-2944b*	TATCACAGCAGTAGTTACCTGA	CONTIG_18252	93731	93792	+	24	0	0
aae-miR-N1-1	aae-miR-2941-2	TAGTACGGCTAGAACTCCACGGA	CONTIG_16241	318005	318105	-	288	1	1
aae-miR-N1-2	aae-miR-2941-1	TAGTACGGCTAGAACTCCACGGA	CONTIG_16241	317700	317799	-	288	1	1
aae-miR-N2	aae-miR-2946	TAGTACGGAAAAGATATGGGGA	CONTIG_16241	318135	318222	-	5	0	0
									
aae-miR-1174	aae-miR-1174	TCAGATCTACTTAATACCCAT	CONTIG_7232	17531	17665	+			
aae-miR-1175	aae-miR-1175	TGAGATTCTACTTCTCCGACTTAA	CONTIG_7232	17737	17816	+	0	0	1
aae-miR-1175*	aae-miR-1175*	TAAGTGGAGTAGTGGTCTCATCGCT	CONTIG_7232	17737	17816	+	0	2	59
aae-miR-1890	aae-miR-1890	TGAAATCTTTGATTAGGTCT*GG*	CONTIG_10435	60786	60931	+	1	0	0
aae-miR-1891-1	aae-miR-1891-1	TGAGGAGTTAATTTGCGTGTTT	CONTIG_10244	3984	4069	-	2	0	0
aae-miR-1891-2	aae-miR-1891-2	TGAGGAGTTAATTTGCGTGTTT	CONTIG_18287	4656	4741	+	2	0	0
aae-miR-1889	aae-miR-1889	CACGTTACAGATTGGGGTTTCC	CONTIG_4323	23786	23921	-	0	0	3
									
aae-bantam	aae-bantam	TGAGATCATTTTGAAAGCTGATT	CONTIG_3301	208288	208426	-	1	2	2
aae-let-7	aae-let-7	TGAGGTAGTTGGTTGTATAGT	CONTIG_2929	321614	321684	+	0	0	5
aae-miR-1	aae-miR-1	TGGAATGTAAAGAAGTATGGAG	CONTIG_25061	66256	66338	+	1	0	1
aae-miR-10	aae-miR-10	ACCCTGTAGATCCGAATTTGTT	CONTIG_17639	8749	8833	+	1	0	0
aae-miR-100	aae-miR-100	AACCCGTAGATCCGAACTTGTG	CONTIG_2929	307441	307568	+			
aae-miR-1000-1	aae-miR-1000-1	ATATTGTCCTGTCACAGCAGT	CONTIG_35246	172	265	-			
aae-miR-1000-2	aae-miR-1000-2	ATATTGTCCTGTCACAGCAGT	CONTIG_9774	12985	13078	+			
aae-miR-11	aae-miR-11	CATCACAGTCTGAGTTCTTGCTT	CONTIG_23911	46399	46488	+	1861	42	436
aae-miR-11*	aae-miR-11*	CGAGAACTCCGGCTGTGACCTGTG	CONTIG_23911	46399	46488	+	4	0	2
aae-miR-12	aae-miR-12	TGAGTATTACATCAGGTACTGGT	CONTIG_4323	23386	23503	-			
aae-miR-124	aae-miR-124	TAAGGCACGCGGTGAATGCCAAG	CONTIG_531	21343	21423	-	3	0	0
aae-miR-125	aae-miR-125	TCCCTGAGACCCTAACTTGTGA*C*	CONTIG_2929	321885	321975	+	0	0	3
aae-miR-133	aae-miR-133	TTGGTCCCCTTCAACCAGCTGT	CONTIG_24522	50243	50347	-			
aae-miR-137-1	aae-miR-137-1	TTATTGCTTGAGAATACACGTA	CONTIG_7773	15414	15486	-			
aae-miR-137-2	aae-miR-137-2	TTATTGCTTGAGAATACACGTA	CONTIG_29705	92457	92529	+			
aae-miR-13b	aae-miR-13	TATCACAGCCATTTTGACGAGTT	CONTIG_12793	17619	17710	-	168	2	42
aae-miR-14	aae-miR-14	TCAGTCTTTTTCTCTCTCCTAT	CONTIG_12112	693	785	-	1	1	6
aae-miR-184	aae-miR-184	TGGACGGAGAACTGATAAGGGC	CONTIG_19030	3452	3535	-	957	76	1307
aae-miR-190	aae-miR-190	AGATATGTTTGATATTCTTGGTTG	CONTIG_10108	2092	2180	-	83	2	43
aae-miR-193	aae-miR-193	TACTGGCCTACTAAGTCCCAAC	CONTIG_19568	51035	51158	+			
aae-miR-2a	aae-miR-2a	TATCACAGCCAGCTTTGATGAGCT	CONTIG_12793	16130	16216	-	1217	80	249
aae-miR-210	aae-miR-210	TTGTGCGTGTGACAACGGCTAT	CONTIG_19443	85221	85292	-	3	12	12
aae-miR-219	aae-miR-219	TGATTGTCCAAACGCAATTCTTG	CONTIG_15776	56993	57078	+			
aae-miR-2b	aae-miR-2b	TATCACAGCCAGCTTTGAAGAGCG	CONTIG_12793	17471	17562	-	758	74	147
aae-miR-2c	aae-miR-2c	TATCACAGCCAGCTTTGATGAGC	CONTIG_12793	17971	18048	-	1218	80	249
aae-miR-252	aae-miR-252	CTAAGTACTAGTGCCGCAGGAGA	CONTIG_3685	79002	79177	-	38	0	1
aae-miR-252*	aae-miR-252*	CCTGCTGCCCAAGTGCTTATCGAA	CONTIG_3685	79002	79177	-	5	0	0
aae-miR-263	aae-miR-263a	AATGGCACTGGAAGAATTCACGGG	CONTIG_27430	4746	4821	-	53	1	1
aae-miR-263*	aae-miR-263a*	CGTGTTCTGGCAGTGGCATCCC	CONTIG_27430	4746	4821	-	6	0	0
aae-miR-263b	aae-miR-263b	CTTGGCACTGGGAGAATTCACAG	CONTIG_1908	33267	33357	+	6	0	0
aae-miR-263b*	aae-miR-263b*	TGGATCTTTTCGTGCCATCGT	CONTIG_1908	33267	33357	+	1	0	0
aae-miR-275	aae-miR-275	TCAGGTACCTGAAGTAGCGCGCG	CONTIG_1651	96625	96720	+	0	0	5
aae-miR-275*	aae-miR-275*	CGCGCTAAGCAGGAACCGAGACT	CONTIG_1651	96625	96720	+	1	0	6
aae-miR-276-1	aae-miR-276-1	TAGGAACTTCATACCGTGCTCT	CONTIG_417	10988	11177	-			
aae-miR-276-2	aae-miR-276-2	TAGGAACTTCATACCGTGCTCT	CONTIG_7617	38953	39087	+			
aae-miR-277	aae-miR-277	TAAATGCACTATCTGGTACGACA	CONTIG_12646	3556	3644	+			
aae-miR-278	aae-miR-278	TCGGTGGGACTTTCGTCCGTTT	CONTIG_1172	33037	33128	+	6	0	2
aae-miR-279	aae-miR-279	TGACTAGATCCACACTCATTAA	CONTIG_17556	116934	117006	-	26	0	2
aae-miR-281	aae-miR-281	CTGTCATGGAATTGCTCTCTTTA	CONTIG_27100	34036	34204	+	1	16	50
aae-miR-281*	aae-miR-281*	AAAGAGAGCTATCCGTCGACAGTA	CONTIG_27100	34036	34204	+	164	5686	4806
aae-miR-282-1	aae-miR-282-1	AATCTAGCCTCTCCTAGGCTTTGTCTG	CONTIG_15805	19414	19548	+			
aae-miR-282-1*	aae-miR-282-1*	ACATAGCCTGACAGAGGTTAGG	CONTIG_15805	19414	19548	+	2	0	0
aae-miR-282-2	aae-miR-282-2	AATCTAGCCTCTCCTAGGCTTTGTCTG	CONTIG_15982	5479	5613	-			
aae-miR-282-2*	aae-miR-282-2*	ACATAGCCTGACAGAGGTTAGG	CONTIG_15982	5479	5613	-	2	0	0
aae-miR-283	aae-miR-283	CAATATCAGCTGGTAATTCTGG*G*	CONTIG_4323	32334	32426	-	6	1	131
aae-miR-285	aae-miR-285	TAGCACCATTCGAAATCAGT	CONTIG_1634	212427	212492	-	0	1	0
aae-miR-286a-1	aae-miR-286b-1	TGACTAGACCGAACACTCGTATCC	CONTIG_21241	18407	18503	+	8	0	0
aae-miR-286a-2	aae-miR-286b-2	TGACTAGACCGAACACTCGTATCC	CONTIG_8291	17656	17752	+	8	0	0
aae-miR-286b	aae-miR-286a	TGACTAGACCGAACACTCGCGTC*CT*	CONTIG_18252	93413	93509	+	10	0	0
aae-miR-305	aae-miR-305	ATTGTACTTCATCAGGTGCTCTGG	CONTIG_1651	105677	105766	+	0	0	2
aae-miR-305*	aae-miR-305*	CGGCACATGTTGGAGTACACTTAA	CONTIG_1651	105677	105766	+	0	0	7
aae-miR-306	aae-miR-306	TCAGGTACTGAGTGACTCTCAG	CONTIG_24640	55004	55128	+	119	0	27
aae-miR-307	aae-miR-307	TCACAACCTCCTTGAGTGAGCGA	CONTIG_1157	147943	148043	-	1	0	0
aae-miR-308	aae-miR-308	AATCACAGGAGTATACTGTGAG	CONTIG_6324	6462	6528	+			
aae-miR-308*	aae-miR-308*	CGCGGTATATTCTTGTGGCTTGA	CONTIG_6324	6462	6528	+	2	0	0
aae-miR-31	aae-miR-31	TGGCAAGATGTTGGCATAGCTGAAA	CONTIG_21960	98153	98301	-	1	0	3
aae-miR-315	aae-miR-315	TTTTGATTGTTGCTCAGAAAGCC	CONTIG_21501	12498	12562	+	27	1	0
aae-miR-315*	aae-miR-315*	CTTTCGAGCAGTAATCAAAGTc	CONTIG_21501	12498	12562	+	5	0	0
aae-miR-316	aae-miR-316	TGTCTTTTTCCGCTTACTGCCG	CONTIG_13460	10738	10828	-	0	0	1
aae-miR-317-1	aae-miR-317-1	TGAACACAGCTGGTGGTATCTCAGT	CONTIG_12640	88437	88524	+	36	38	737
aae-miR-317-2	aae-miR-317-2	TGAACACAGCTGGTGGTATCTCAGT	CONTIG_8451	9550	9637	-	36	38	737
aae-miR-3a-1	aae-miR-309a-1	TCACTGGGCAAAGTTTGTCGCA	CONTIG_21241	18967	19043	+	6	0	0
aae-miR-3a-2	aae-miR-309a-2	TCACTGGGCAAAGTTTGTCGCA	CONTIG_8291	18216	18292	+	6	0	0
aae-miR-3b	aaw-miR-309b	TCACTGGGCATAGTTTGTCGCA	CONTIG_18252	94047	94117	+	3	0	0
aae-miR-3b*	aae-miR-309b*	CGTCAAACTCCGTTCAGTTGGTG	CONTIG_18252	94047	94117	+	1	0	0
aae-miR-33	aae-miR-33	GTGCATTGTAGTTGCATTGCA	CONTIG_18815	108787	108866	+			
aae-miR-34	aae-miR-34	TGGCAGTGTGGTTAGCTGGTTGTG	CONTIG_12646	4278	4400	+	44	55	639
aae-miR-375	aae-miR-375	TTTGTTCGTTTGGCTCGAGTTA	CONTIG_14081	238834	238944	-	1	0	0
aae-miR-7	aae-miR-7	TGGAAGACTAGTGATTTTGTTGTT	CONTIG_31115	46028	46112	+	14	0	0
aae-miR-71	aae-miR-71	AGAAAGACATGGGTAGTGAGATA	CONTIG_12793	18215	18396	-	39	2	7
aae-miR-71*	aae-miR-71*	TCTCACTACCTTGTCTTTCATG	CONTIG_12793	18215	18396	-	6	0	0
aae-miR-79	aae-miR-79	ATAAAGCTAGATTACCAAAGCAT	CONTIG_24640	55215	55284	+	2	0	0
aae-miR-8	aae-miR-8	TAATACTGTCAGGTAAAGATGTC	CONTIG_16942	34594	34660	+	32	1	38
aae-miR-8*	aae-miR-8*	CATCTTACCGGGCAGCATTAGA	CONTIG_16942	34594	34660	+	7	2	4
aae-miR-87	aae-miR-87	GGTGAGCAAATTTTCAGGTGT	CONTIG_15587	30157	30251	+			
aae-miR-927	aae-miR-927	TTTAGAATTCCTACGCTTTACC	CONTIG_1795	170460	170534	+			
aae-miR-929-1	aae-miR-929-1	ATTGACTCTAGTAGGGAGTCC	CONTIG_12461	76647	76734	-			
aae-miR-929-2	aae-miR-929-2	ATTGACTCTAGTAGGGAGTCC	CONTIG_34315	3294	3381	+			
aae-miR-92a	aae-miR-92a	TATTGCACTTGTCCCGGCCTAT	CONTIG_6821	67755	67832	+	74	0	5
aae-miR-92a*	aae-miR-92a*	CGGTACGGACAGGGGCAACATT	CONTIG_6821	67755	67832	+	6	0	0
aae-miR-92b	aae-miR-92b	AATTGCACTTGTCCCGGCCTGC	CONTIG_6824	4049	4131	+	74	0	5
aae-miR-92b*	aae-miR-92b*	AGGTCGTGACTTGTGCCCGTTTG	CONTIG_6824	4049	4131	+	12	0	0
aae-miR-932	aae-miR-932	TCAATTCCGTAGTGCATTGCAG	CONTIG_28378	72232	72319	-			
aae-miR-957	aae-miR-957	TGAAACCGTCCAAAACTGAGGC	CONTIG_591	147619	147787	+			
aae-miR-965	aae-miR-965	TAAGCGTATAGCTTTTCCC	CONTIG_3410	87378	87524	+			
aae-miR-970	aae-miR-970	TCATAAGACACACGCGGCTAT	CONTIG_11324	29252	29338	+	1	0	3
aae-miR-981	aae-miR-981	TTCGTTGTCGACGAAACCTGCA	CONTIG_7302	184768	184853	-			
aae-miR-988	aae-miR-988	CCCCTTGTTGCAAACCTCACGC	CONTIG_17684	77966	78086	-	3	0	1
aae-miR-988*	aae-miR-988*	GTGTGCTTTGTGACAATGAGA	CONTIG_17684	77966	78086	-	2	0	2
aae-miR-989	aae-miR-989	TGTGATGTGACGTAGTGGTAC	CONTIG_6774	51048	51137	-	2	33	3
aae-miR-993	aae-miR-993	GAAGCTCGTTTCTATAGAGGTATCT	CONTIG_28305	82579	82660	+			
aae-miR-996	aae-miR-996	TGACTAGATTACATGCTCGTCT	CONTIG_17556	112174	112267	-			
aae-miR-998	aae-miR-998	TAGCACCATGAGATTCAGCTC	CONTIG_23911	46676	46764	+	164	6	42
aae-miR-999	aae-miR-999	TGTTAACTGTAAGACTGTGTCT	CONTIG_6027	163128	163269	+			
aae-miR-9a-1	aae-miR-9a-1	TCTTTGGTTATCTAGCTGTATGA	CONTIG_19539	116871	116951	+	16	1	2
aae-miR-9a-2	aae-miR-9a-2	TCTTTGGTTATCTAGCTGTATGA	CONTIG_19541	4061	4141	+	16	1	2
aae-miR-9b	aae-miR-9b	TCTTTGGTGATTTTAGCTGTATGC	CONTIG_24640	55513	55604	+	134	0	69
aae-miR-9c	aae-miR-9c	TCTAAAGCTTTAGTACCAGAGGTC	CONTIG_24640	28160	28257	+	5	0	0
aae-miR-iab-4-1	aae-miR-iab-4-1	CGGTATACCTTCAGTATACGTAAC	CONTIG_17255	44653	44726	-			
aae-miR-iab-4-2	aae-miR-iab-4-2	CGGTATACCTTCAGTATACGTAAC	CONTIG_23219	23581	23654	+			

Notes:

**1**. The first block (miR-M1-1 through miR-N2) are novel miRNAs that are discovered in this study. We have not detected homology to any known miRNAs or genomic sequences outside of mosquito species. The second block (miR-1174 through miR-1889) contains miRNAs that are homologous to previously reported "mosquito-specific" miRNAs. The third block, comprised of the remaining miRNAs, contains miRNAs that have homologues outside of mosquitoes.

**2**. Naming in this column is temporary. Mirbase assigned names are shown in column 2, which was received after the acceptance of the manuscript. "-1", and "-2" suffixes refer to different hairpins that produce the same mature miRNA. "a", and "b" suffixes refer to different hairpins that produce similar but not identical miRNAs.

**3**. Underlined sequences are cases where there are extra bases at the 5' or 3' ends and they are the majority in at least six small RNA sequences. Italicized sequences are cases where such sequences are detected but they are either not the majority or there are less than six 454 sequence hits. As poly-As are added to the small RNAs, adenine(s) at the 3' end cannot be confirmed by this sequencing approach.

**4**. The contig, start, and end positions refer to the locations of the pre-miRNA hairpins.

**5**. The last three columns are the number of sequence hits in small RNA libraries obtained by 454 sequencing. The total small RNA reads are 55, 000, 33, 000, and 42, 000 in embryos, sugar-fed midguts (Gut_SF), and blood-fed midguts (Gut_BF), respectively. The total number of hits for all *Ae. aegypti *miRNAs are 8369, 6260, and 9922 in embryos, sugar-fed midguts (Gut_SF), and blood-fed midguts (Gut_BF), respectively. There are a few cases where we did not distinguish the hits from nearly identical miRNAs.

### Possible revisions at the ends of known miRNAs and cases of abundant miRNA* sequences

As shown in Table 1, there are a few cases where the *Ae. aegypti *miRNA sequences, as indicated by direct sequencing, start or end with one or a few extra nucleotides compared to the known miRNAs reported from *D. melanogaster *or anopheline mosquitoes (miRBase). To minimize the contribution of sequencing error, we only consider cases where there are at least six such sequences in the 454 database and these differences are the majority. These miRNAs include aae-miR-2a, aae-miR-210, aae-miR-263b, aae-miR-281, and aae-miR-283. Because internal sequence variations between miRNAs from different species could simply result from species differences, we did not include in the above list the aae-miRNAs that only had internal sequence variations compared to known miRNAs. On the other hand, shifts at the 5' or 3' ends could either suggest a difference between species or imprecise annotation at the miRNA termini. Thus the above 5 aae-miRNAs provide leads to further studies to investigate whether these previously reported miRNA sequences need to be revised.

In vast majority of the cases, mature miRNAs are much more abundant than miRNA*. However, miR-281* and miR-1175* are at least a few dozen fold more abundant than their miRNA sequences. In both cases, the miRNA and the miRNA* sequences are 100% identical among *Ae. aegypti*, *Cx. quinquiefasciatus*, and *An. gambiae*. It is therefore possible that miR-281* and miR-1175* are functional. There are a few other cases in which the miRNA* is more abundant than the miRNA sequences (Table 1). However, the numbers of hits are low in these cases and it is difficult to assess how significant the differences may be.

### miRNA gene clusters and duplications: evolutionary implications

There are 14 clusters of miRNAs that are defined as more than one miRNA hairpin within 10 kb [29]. Twelve of these clusters have members that are separated by less than one kb. All these clusters can be identified in Table 1 and Additional file 1 by tracking and sorting the contigs and start and end positions of the pre-miRNAs. Two previously identified clusters are worth noting. The first is the cluster that includes miR-9b, miR-79, and miR-306. We previously identified this cluster in both *An. gambiae *and *D. melanogaster *and we thought miR-306 was missing in the *Ae. aegypti *cluster [27]. However, small RNA sequencing and closer analysis of the *Ae. aegypti *assembly suggest that miR-306 is indeed present in *Ae. aegypti *and the relative positions of the three miRNAs in the cluster are conserved among all three species. The aae-miR-306 shows 2 mismatches in the 22 bp overlap compared to aga-miR-306 and dme-miR-306.

The second cluster includes miR-12 and miR-283, which flank either miR-304 in *D. melanogaster *or miR-1889 in *An. gambiae *[27]. MiR-1889 has similarity to the reverse-complementary sequence of miR-304 but the difference is significant enough for miRbase to assign a unique name for it. Given that the *D. melanogaster *miR-304 and the *An. gambiae *miR-1889 are flanked by the same miRNAs and that this miRNA cluster is found in an intron of orthologous genes in the two species, miR-304 and miR-1889 may have a common origin. Through small RNA sequencing and a closer analysis of the genome assembly, we identified miR-1889 in *Ae. aegypti *(Table 1 and Additional file 1), which was previously thought to be missing in the *Ae. aegypti *cluster [27]. The newly identified aae-miR-1889 shows 3 mismatches in the 21 bp overlap with aga-miR-1889. The identification of aae-miR-1889 further supports the strand orientation of the mosquito miR-1889. It is tempting to suggest that one of the two miRNA hairpins, miR-304 or miR-1889, was inverted during evolution. It is also possible that fruit flies and mosquitoes utilize different strands of the hairpin as mature miRNA. Wecurrently do not have evidence to support either of these hypotheses.

There are 17 cases where one of the pre-miRNAs is duplicated and thus more than one pre-miRNA hairpin produces the same or highly similar mature miRNAs. These pre-miRNAs are shown either with a suffix of "-1" and "-2" for hairpins that produce identical miRNAs or with a suffix of "a" or "b" for hairpins that produce highly similar miRNAs (Table 1). These miRNAs are a rich source for future comparative analysis to uncover the evolutionary patterns of miRNA duplication and the process of creating novel miRNAs in mosquitoes. It remains to be determined whether the rather common miRNA duplication observed in *Ae. aegypti *reflect the importance of duplication for the generation of new miRNAs in mosquitoes. In this regard, it is interesting to note that while duplication is a common mechanism to generate new miRNAs in plants (e.g., [30]), duplication was thought to be not important in *Drosophila *[3].

### Novel miRNAs that are potentially specific to mosquitoes

**Figure 1:**
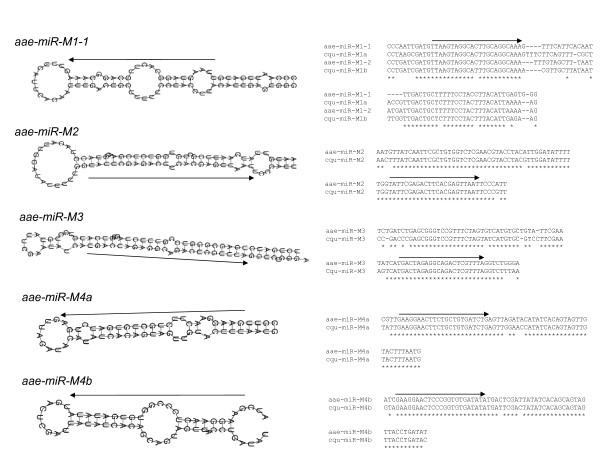
**Alignments and stem-loop structures of five novel mosquito pre-miRNAs**. See Table 1 for naming and sequence locations of these miRNAs. Left panels are the hairpin structures. Right panels are the sequence alignments between *Ae. aegypti *miRNAs (aae-miRNAs) and *Cx. quinquefasciatus *miRNAs (cqu-miRNAs). Arrows point to the mature miRNA sequences from 5' to 3'. In the case of miR-M1, there are two physically linked copies in both *Ae. aegypti *and *Cx. quinquefasciatus*, as shown in the alignment and in Table 1. The two copies in *Ae. aegypti *produce the same mature miRNAs and the hairpins are named -1 and -2. The two copies in *Cx. quinquefasciatus *are named -1a and -1b because their mature miRNAs differ by one nucleotide. Only the hairpin structure for aae-miR-M1-1 is shown. All five miRNAs shown in panel A have homologs in *An. gambiae*.

**Figure 2:**
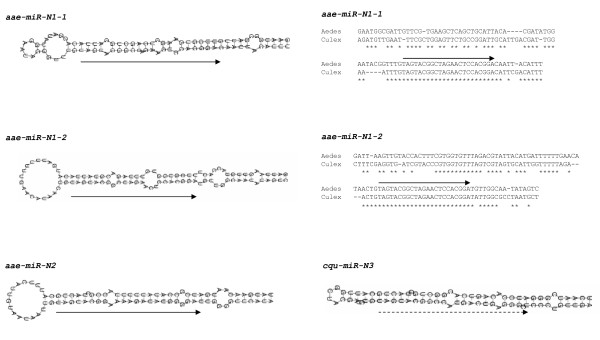
**Alignments and stem-loop structures of a novel miRNA cluster within the intron of a gene encoding a transcription factor**. See Table 1 for naming and sequence locations of these miRNAs. There are two hairpins for the same miR-N1 (-1 and -2) in both *Ae. aegypti *and *Cx. quinquefasciatus*. Only the *Ae. aegypti *hairpin structures for the miR-N1 pre-miRNAs are shown. Aae-miR-N2 and cqu-miR-N3 are only found in *Ae. aegypti *and *Cx. quinquefasciatus*, respectively. Thus there are no alignments for these two miRNAs. Arrows point to the mature miRNA sequences from 5' to 3'. Dashed arrow for cqu-miR-N3 reflects the fact that we do not yet have the direct sequence for this miRNA. The mature cqu-miR-N3 sequence was predicted according to the conserved seed sequence shared with miR-N1 and miR-N2, which was confirmed by northern blots using anti cqu-miR-N3 as a probe (see Figure 7).

Nine of the 98 pre-miRNA hairpins are novel and currently have only been found in mosquitoes. These nine pre-miRNAs produce seven distinct mature miRNAs (miR-M1, -M2, -M3, -M4a and -M4b; miR-N1 and miR-N2). All seven mature miRNAs have multiple hits from small RNA sequencing, confirming their status as miRNAs. A few of these also have hits in the miRNA* strand. Shown in Figures 1 and 2 are the sequence alignments of the novel pre-miRNA sequences discovered in this study and the hairpins they form. Two physically linked pre-miRNA hairpins (miR-M1-1 and miR-M1-2) produce the same miR-M1 in *Ae. aegypti*. Two physically linked pre-miRNA hairpins produce similar but not identical miR-M4a and miR-M4b. MiR-M1, -M2, -M3, -M4a and -M4b are found in all three available mosquito genome assemblies.

Two physically linked pre-miRNA hairpins (miR-N1-1 and miR-N1-2) produce the same miR-N1 and they are also in close proximity to the miR-N2 hairpin in *Ae. aegypti*. The three hairpins are in the first intron of a gene in *Ae. aegypti *(Vectorbase Gene ID AAEL009263) encoding a putative transcription factor with a basic leucine zipper domain. Sequence analysis suggests that miR-N1 is found in the orthologous gene in *Cx. quinquefasciatus *but not found in *An. gambiae*. MiR-N2 is only found in *Ae. aegypti*. MiR-N1 also exists in two hairpins in the intron of the homologous gene in *Cx. quinquefasciatus*and there is a third hairpin that has a predicted miRNA with a similar 5' sequence as miR-N1. We name this miRNA miR-N3 and it is only found in *Cx. quinquefasciatus *(Vectorbase Gene ID CPIJ000468). Cqu-miR-N3 is not listed in Table 1, which only shows miRNAs from *Ae. aegypti*.

Thus, we have identified eight novel mosquito-specific miRNAs in this study. We define "mosquito-specific" miRNAs here as those that are currently only found in mosquitoes. BLAST searches using low stringent parameters (word size at seven, e-value cut-off at 10) failed to identify any reliable homologues from miRBase or non-redundant GenBank sequences. We also performed oligomap comparisons [31] of the "mosquito-specific" miRNAs to all miRBase sequences using default parameters and did not identify any match in any other organism. Oligomap [31] is designed for comparisons of short sequences such as miRNAs, allowing gaps and mismatches. Taken together, the evidence indicates that what we are reporting in this study are novel miRNAs. This study increased the number of novel miRNAs that are only found in mosquitoes from five [21,27] to 13. It is important to emphasize that some of these so-called "mosquito-specific" miRNAs may be discovered outside mosquitoes as future efforts of genome and small RNA sequencing expand to more and more organisms.

### Expression patterns of conserved miRNAs

**Figure 3:**
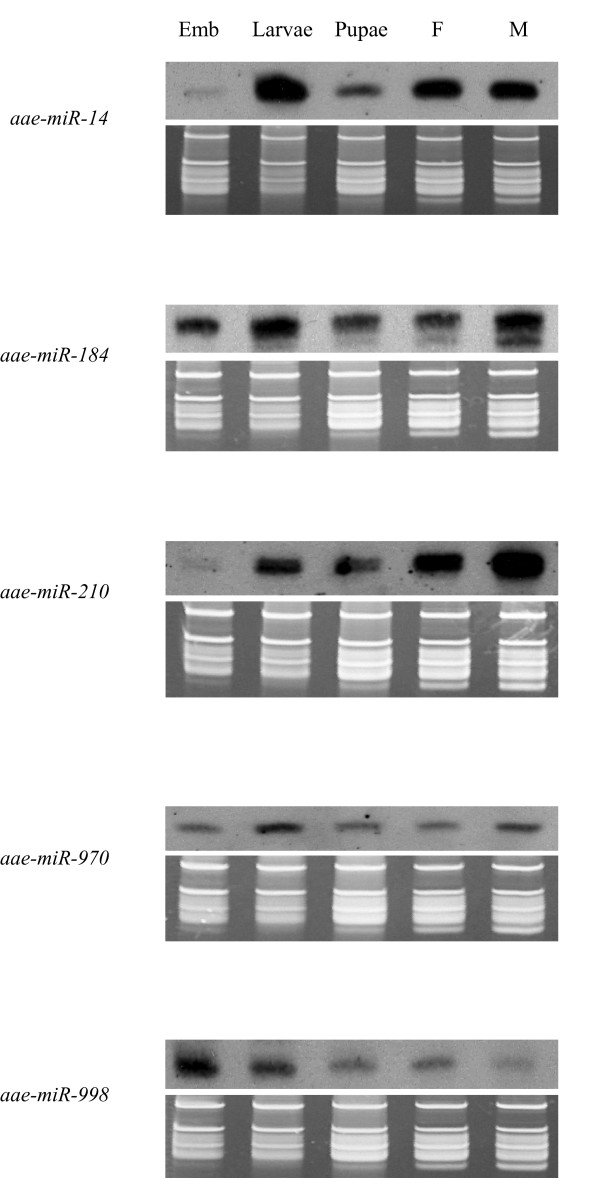
**Expression patterns of *Ae. aegypti *homologs of previously known miRNAs**. Only *Ae. aegypti *RNA samples were examined. The top panels are northern results and the bottom panels are RNA gel images for verification of small ribosomal RNA and tRNA integrity and loading of total RNA. Emb, pooled embryos between 0-36 hr after egg deposition; Larvae; mixed instar larvae; Pupae, mixed puape; F, adult females one to five days after emergence; M, adult males one to five days after emergence. 10 μg of total RNA were used per sample.

We chose nine conserved miRNAs and eight mosquito-specific miRNAs for further analysis using northern blot to confirm the small RNA sequencing results and to determine the expression patterns of these miRNAs in different developmental stages. Expression analysis of the eight mosquito-specific miRNAs is described in the context of a multi-species survey in a later section. The nine conserved miRNAs include let-7, miR-1, -133, -14, -184, -210, -9a, -970, and -998. All nine miRNAs showed signals at ~20 nt by northern during at least one of the developmental stages. Shown in Figure 3 are the expression patterns of five of the nine conserved miRNAs. The patterns of presence/absence of these miRNAs in embryo, larvae, pupa, and adult stages are similar to the patterns found in *D. melanogaster *[32] and *An. stephensi *[27].

### Elevated levels of miRNAs after blood feeding in the midgut of *Ae. aegypti*

**Figure 4:**
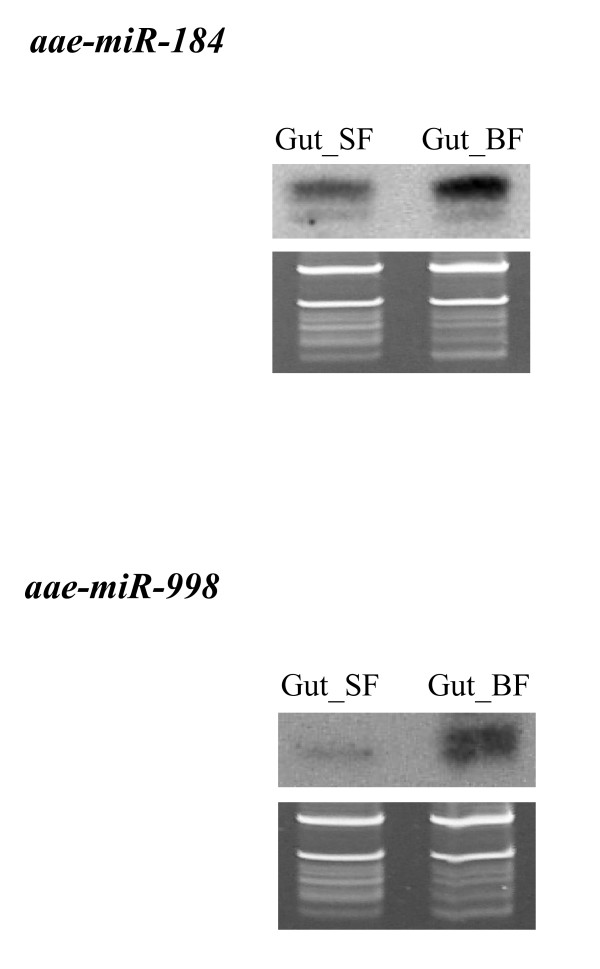
**Higher levels of miRNAs are observed in the female *Ae. aegypti *midgut 24 hrs after blood feeding (Gut_BF) compared to sugar feeding (Gut_SF)**. Three-day old females were either fed on blood or sugar and dissected 24 hrs later. 10 μg of total RNA were used per sample. The top panels are northern results and the bottom panels are RNA gel images for verification of small ribosomal RNA and tRNA integrity and loading of total RNA.

The numbers of small RNA sequences in the midgut samples from sugar-fed and blood-fed female *Ae. aegypti *may not be high enough for quantitative comparison. Nonetheless, we decided to compare the relative miRNA levels for miRNAs that showed more than 25 hits in at least one of the midgut samples. We used either the total number of all miRNA hits [12] or the total number of small RNA reads to normalize the data. As shown in the last two columns of Table 2, except for miR-989 and miR-281*, all miRNAs showed an increase after blood feeding. We then performed northern blots using miR-184 and miR-998 probes. It is clear that miR-998 level is higher in bloodfed samples than in sugar-fed samples. Although less obvious, miR-184 level also appears to be higher in blood-fed samples than in sugar-fed samples (Figure 4). Thus, the northern results are largely consistent with the data shown in Table 1. We have previously analyzed the level of miR-989 in the midgut before and after blood feeding [27]. The signal was too weak to confirm or rule out reduction of miR-989 after blood feeding. Some miRNAs that are expressed in the midgut samples are also found in large numbers in embryos in *Ae. aegypti *(Table 1).

**Table 2:** Comparison of the number of miRNA sequences in sugar-fed and blood-fed midgut samples.

**miRNAs **^**1**^	**Gut_SF **^**6**^	**Gut_BF **^**6**^	**Fold Change I **^**7, 9**^	**Fold Change II **^**8, 9**^
aae-miR-11 ^2^	42	436	6.55	8.16
aae-miR-1175*	2	59	18.61	23.18
aae-miR-13b ^3^	2	42	13.25	16.50
aae-miR-184	76	1307	10.85	13.51
aae-miR-190	2	43	13.56	16.89
aae-miR-281	16	50	1.97	2.46
aae-miR-281*	5686	4806	0.53	0.66
aae-miR-283	1	131	82.65	102.93
aae-miR-2a/2b/2c ^3, 4^	80	249	1.96	2.45
aae-miR-306 ^5^	0	27	NA ^10^	NA ^10^
aae-miR-317-1	38	737	12.24	15.24
aae-miR-317-2	38	737	12.24	15.24
aae-miR-34	55	639	7.33	9.13
aae-miR-8	1	38	23.98	29.86
aae-miR-989	33	3	0.06	0.07
aae-miR-998 ^2^	6	42	4.42	5.50
aae-miR-9b 5	0	69	NA ^10^	NA ^10^

Notes.

1. Only miRNAs that showed 25 or more sequences in one of the gut samples are shown.

2. MiR-11 and miR-998 are physically linked, less than 300 bp apart.

3. MiR-13b and miR-2a/2b/2c are in a physically linked cluster. Mir-13b is less than 200 bp apart from miR-2b.

4. It is difficult to distinguish between hits that match miR-2a, miR-2b, or miR-2c. Thus we refer to these hits miR-2a/2b/2c.

5. MiR-306 and miR-9b are physically linked, less than 500 bp apart.

6. The second and third columns are raw numbers of sequence hits in sugar-fed (Gut_SF) and blood-fed (Gut_BF) midgut samples, respectively.

7. Column four is the fold difference of Gut_BF over Gut_SF, normalized by the total miRNA hits within a sample, as suggested in reference [12]. The total miRNA hits are 6260 and 9922 in Gut_SF and Gut_BF samples, respectively. Thus, for each miRNA, the Fold Change = [(Raw Gut_BF number)/9922]/[(Raw Gut_SF number)/6260].

8. Column five is the fold difference of Gut_BF over Gut_SF, normalized by the total small RNA reads within a sample. The total small RNA reads are 33, 000 and 42, 000 in Gut_SF and Gut_BF samples, respectively. Thus, for each miRNA, the Fold Change = [(Raw Gut_BF number)/42000]/[(Raw Gut_SF number)/33000].

9. The numbers in columns 4 and 5 reflect the same trend, generally higher levels of these miRNAs in blood-fed sample than in sugar-fed sample. Although the number of small RNA sequences may not be sufficient for the analysis to be quantitative, overall trends suggest leads for further analysis.

10. NA, not applicable as the denominator is zero.

Blood feeding is critical for mosquito physiology and its ability to transmit disease pathogens. It is through feeding on an infected host mosquitoes acquire pathogens such as malaria parasites and dengue viruses. It is also through blood feeding by an infected mosquito these pathogens may spread to a different host. Midgut is the first barrier the pathogens have to cross before they establish infection in mosquitoes. Thus midgut is one of the most important links in the disease transmission cycle. Furthermore, blood-feeding triggers a cascade of gene regulatory events in multiple tissues including midgut through the interplay of endocrine signals and transcription factors and thus has great impact on mosquito biology [33-36]. The correlation between blood feeding and miRNA levels in *Ae. aegypti *midgut warrants further investigations, which may shed light on the possible roles of miRNAs in physiology related to blood feeding and perhaps in mosquito-pathogen interactions.

### Multi-species survey of eight mosquito-specific miRNAs revealed conserved and lineage-specific miRNAs

Previously five miRNAs were reported to be only found in mosquitoes. These are miR-1174, miR-1175 [21], miR-1889, miR-1890, and miR-1891 [27]. As described above, we uncovered eight additional mosquito miRNAs, bringing the number of total "mosquito-specific" miRNAs to 13. We conducted a detailed multi-species expression analysis of eight of the 13 mosquito-specific miRNAs using northern blot. When appropriate, we examined the expression of these miRNAs across the life stages of four mosquito species, *An. gambiae, An. stephensi, Ae. aegypti*, and *T. amboinensis*.

**Figure 5:**
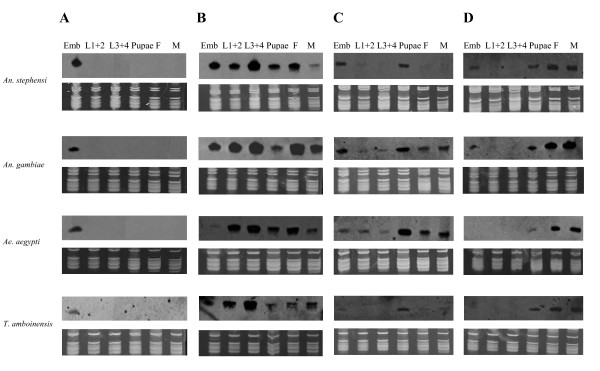
**Four mosquito-specific miRNAs that are expressed in all four species of three highly divergent genera**. MiRNAs examined include miR-M1 (A), miR-1175 (B), miR-1890 (C), and miR-1891 (D). Expression was examined across the developmental stages of *An. stephensi, An. gambiae, Ae. aegypti*, and *T. amboinensis*. The top panels are northern results and the bottom panels are RNA gel images for verification of small ribosomal RNA and tRNA integrity and loading of total RNA. Emb, pooled embryos between 0-36 hr after egg deposition; L1+2, pooled 1^st ^and 2^nd ^instar larvae; L3+4, pooled 3^rd ^and 4^th ^instar larvae; Pupae, mixed pupae; F, adult females one to five days after emergence; M, adult males 1-5 days after emergence. 15 μg of total RNA per sample for *An. stephensi, An. gambiae*, and *Ae. aegypti *were used. 10 μg of *T. amboinensis *total RNA per sample were used. For *T. amboinensis *northerns, 3^rd ^instar larvae were not included. For the *T. amboinensis *miR-M1 northern, hybridization and washes were carried out at 49°C instead of 42°C to reduce background across the membrane.

Four of the eight miRNAs (miR-M1, -1175, -1890, and -1891) are detected in all of the above four species (Figure 5). Furthermore, the expression patterns of these miRNAs are similar in the four species and expression is detected in multiple developmental stages in three of the four miRNAs. The exceptions are the relatively low embryonic expression of miR-1175 in *Ae. aegypti *(Figure 5B) and the low or hardly detectable embryonic expression of miR-1891 in *Ae. aegypti *and *T. amboinensis *(Figure 5D), compared to the rest of the species studied here. Overall, this is consistent with the observation that conserved miRNAs tend to be widely expressed [12,37]. On the other hand, miR-M1 is expressed only in the embryos in all four species.

**Figure 6:**
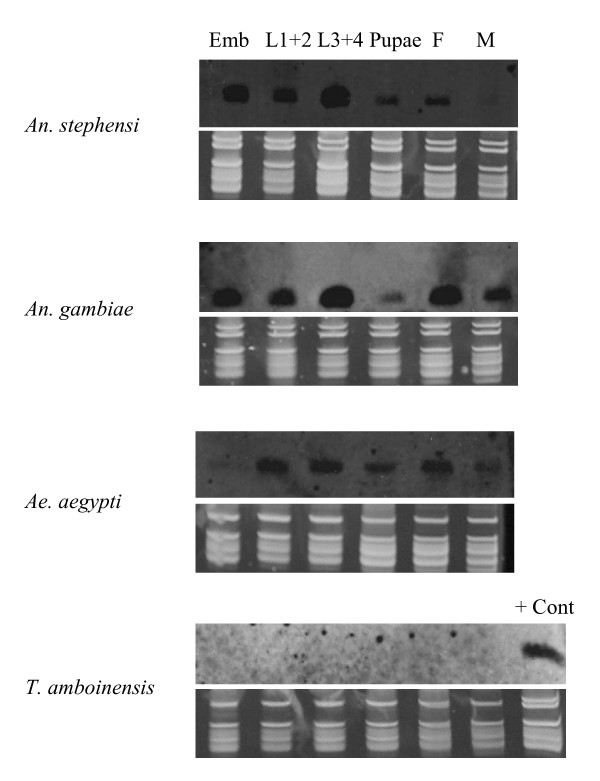
**MiR-1174 is expressed in *An. stephensi, An. gambiae*, and *Ae. aegypti*, but not in *T. amboinensis***. The top panels are northern results and the bottom panels are RNA gel images for verification of small ribosomal RNA and tRNA integrity and loading of total RNA. Emb, pooled embryos between 0-36 hr after egg deposition; L1+2, pooled 1^st ^and 2^nd ^instar larvae; L3+4, pooled 3^rd ^and 4^th ^instar larvae; Pupae, mixed puape; F, adult females one to five days after emergence; M, adult males one to five days after emergence. 15 μg of total RNA per sample for *An. stephensi, An. gambiae*, and *Ae. aegypti *were used. 10 μg of *T. amboinensis *total RNA per sample were used. For *T. amboinensis*, 3^rd ^instar larvae were not included. "+ Cont" indicates a positive control which was *An. stephensi *embryos (12-24 hr).

Four other miRNAs (miR-1174, miR-N1, miR-N2, and miR-N3) are only detected in a subset of the four mosquitoes. As shown in Figure 6, miR-1174 is not found in *T. amboinensis *but strong signals were detected in the other three species. MiR-1174 level in *Ae. aegypti *embryos is low or hardly detectable. It is interesting to point out that miR-1174 and miR-1175 are in the same contig separated by only ~200 bp. The expression patterns of miR-1174 and miR-1175 are similar in all three blood-feeding mosquitoes, suggesting that they may be under the same transcriptional control. MiR-1174 and miR-1175 share some sequence similarity at the 5' end. Thus it is possible that miR-1174 and miR-1175 resulted from gene duplication and miR-1174 may either have been lost in *T. amboinensis *or evolved beyond recognition by the miR-1174 probe. *Ae. aegypti *miR-1174 and *An. gambiae *miR-1174 differ by one nt. It is also possible that miR-1174 simply was not duplicated in *T. amboinensis*.

**Figure 7:**
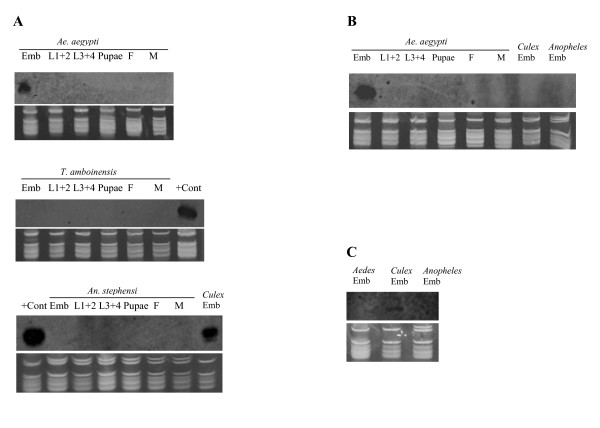
**MiR-N1, miR-N2, and miR-N3 expression is restricted in particular lineages in mosquitoes**. A) miR-N1 is expressed in *Ae. aegypti *and *Cx. quinquefasciatus*, but not in *An. stephensi *nor *T. amboinensis*. Emb, pooled embryos between 0-36 hr after egg deposition; L1+2, pooled 1^st ^and 2^nd ^instar larvae; L3+4, pooled 3^rd ^and 4^th ^instar larvae; Pupae, mixed pupae; F, adult females one to five days after emergence; M, adult males one to five days after emergence. *Culex *Emb, *Cx. quinquefasciatus *embryos 0-24 hrs after egg deposition. "+ Cont", positive control, *Ae. aegypti *embryos (12-24 hr). 15 μg of total RNA per sample for *An. stephensi *and *Ae. aegypti *were used. 10 μg of *T. amboinensis *total RNA per sample were used. For *T. amboinensis*, 3^rd ^instar larvae were not included. B) miR-N2 is expressed in *Ae. aegypti *but not detected in *Cx. quinquefasciatus *and *An. stephensi *embryos. Symbols are as in A. *Anopheles *Emb, pooled *An. stephensi *embryos between 0-36 hr after egg deposition. C) miR-N3 is expressed in *Cx. quinquefasciatus*, but not detected in *Ae. aegypti *and *An. stephensi *embryos. Symbols are as in A and B. *Aedes *Emb, pooled *Aedes aegypti *embryos between 0-36 hr after egg deposition.

As described earlier, miR-N1, N2, and N3 are from the same intronic cluster. As shown in Figure 7, miR-N1 was abundant in both *Ae. aegypti *and *Cx. quinquefasciatus *embryos. It was undetectable in *An. stephensi*. MiR-N2 was abundant in *Ae. aegypti *embryos, but undetectable in the embryos of *Cx. quinquefasciatus*. MiR-N2 was also undetectable in *An. stephensi*. MiR-N3 was found in *Cx. quinquefasciatus *embryos, but not in *Ae. aegypti*. MiR-N3 was also undetectable in *An. stephensi*. The expression data are consistent with genomic sequence analysis, which is described in the previous section on the miR-N1, N2, N3 cluster.

We performed northern blots using all of the above eight miRNA probes to see if any signal was detected in *D. melanogaster*. We used at least 5 μg of total RNA from different developmental stages or a specific stage expected for a particular miRNA. None of the eight probes produced any miRNA signal while the positive control (*Ae. aegypti *sample) showed intense signals (data not shown). This is consistent with these miRNAs being only found in mosquitoes.

### Functions of "mosquito-specific" miRNAs

All of the eight tested mosquito-specific miRNAs showed embryonic expression in at least one mosquito species (Figures 5, 6, and 7), suggesting that these miRNAs may play important roles in mosquito embryonic development. Two of these miRNA clusters are worth noting. The first is the miR-M1-1 and miR-M1-2 cluster, which is only expressed in embryos in all four genera of mosquitoes tested. The conserved expression pattern and sequence conservation across all major branches of Culicidae suggest that miR-M1 is important during mosquito embryogenesis. Another interesting group of miRNAs are the miR-N1, -N2, and -N3 cluster. Two miR-N1 and one miR-N2 hairpins are in the first intron of a gene in *Ae. aegypti *encoding a putative transcription factor. Two miR-N1 hairpins and a miR-N3 hairpin are found in the orthologous gene in *Cx. quinquefasciatus*. None of these miRNAs are found in *An. gambiae*. In addition, miR-N2 is only found in *Ae. aegypti *while miR-N3 exists only in *Cx. quinquefasciatus*. These miRNAs share the same 7-8 bp 5' sequences in the seed regions important for target recognition. MiR-N1, N2, and N3 are all expressed in the embryos. Thus it is possible that these miRNAs derive from duplication events and the duplicated miRNAs may evolve into new sequences that acquire new functions. We postulate that, given their abundance and their lineage specificity, the N1/N2/N3 cluster may play a role in determining important lineage specific traits in mosquitoes. It will be important to determine the targets of these miRNAs to truly understand their function. Currently, the annotation of the 3'-UTRs of *Ae. aegypti *genes is limited. As these annotations improve, miRNA target prediction will likely be fruitful.

## Conclusion

We report the first systematic analysis of miRNAs in *Ae. aegypti *during which 98 pre-miRNAs were uncovered. Thus we substantially expanded the list of miRNAs found in mosquitoes. We also provided experimental evidence for 89 of the miRNA and miRNA* sequences. We also uncovered highly abundant and conserved miRNA* sequences, which is consistent with the suggestion that some miRNA* are functional. There are 14 miRNA clusters and 17 cases where more than one pre-miRNA hairpin produces the same or highly similar mature miRNAs in *Ae. aegypti *. These miRNAs are a rich source for future comparative analysis to uncover the evolutionary patterns of miRNA duplication and the process of creating new miRNAs in mosquitoes. Perhaps most importantly, we identified eight novel miRNAs that are potentially specific to mosquitoes. This discovery expanded the list of mosquito-specific miRNAs from five [21,27] to 13. Four of the 13 miRNAs are specific to certain lineages within mosquitoes. The expression profiles of a few miRNAs suggest stage-specific functions and functions related to embryonic development or blood feeding. A better understanding of the functions of these miRNAs will offer novel insights in mosquito biology and may lead to novel approaches to combat mosquito-borne infectious diseases.

## Methods

### Insects

*Ae. aegypti *(Liverpool strain), *An. gambiae *(G3 strain)*, An. stephensi *(Indian wild type strain), and *T. amboinensis *(CDC strain originally from San Juan, PR)mosquitoes were reared in a humidified insectary at 27°C on a 12 hour light:dark cycle*. Cx. quinquefasciatus *embryos at 0-24 hrs post-egg-deposition were kindly provided by Drs Aaron Brault and David Clark at the University of California, Davis. *D. melanogaster *wild type(Catalina strain, stock number 14021 -0231.47) samples were provided by the Tucson Drosophila Stock Center (Tucson, AZ). *D. melanogaster *W1118 eggs at 0-24 hrs post-egg-deposition were provided by Duke University Model Systems (Duke University, Durham, NC).

### *Ae. aegypti *sample preparation for 454 pyrosequencing

Three samples were prepared for 454 sequencing. Female *Ae. aegypti *were either fed on mice or kept on sugar water, three days post-emergence. Midguts were dissected at 24 hours post blood meal and midguts from sugar-fed females were also dissected at the same time interval. To collect the third sample , which was mixed-age embryos, all eggs were laid on filter papers during one-hour intervals. The filter papers were then kept in an incubator (27°C, ~70% Relative Humidity) before collection at appropriate hours and stored in RNAlater (Ambion, Austin, Texas). The same volumes of eggs from different time periods were then mixed. This collection design ensured that embryos between 0-48 hrs post-egg-deposition were collected in similar quantities. All three samples were sent to vertis Biotechnologie AG (Freising-Weihenstephan, Germany) for small RNA cloning. All samples were ground in liquid nitrogen and RNA smaller than 200 bp were enriched with the mirVana miRNA isolation kit (Ambion). The population of miRNAs with a length of 15-30 bp was passively eluted from polyacrylamide gels. The RNA was then precipitated with ethanol and dissolved in water. The small RNAs collected had a poly(A)-tail added to their 3'-OH by poly-(A) polymerase. The 5'-phosphate of the small RNAs were ligated to an RNA adapter. First-strand cDNA synthesis was then performed using an oligo(dT)-linker primer and MMLV-RNase H reverse transcriptase. The resulting cDNAs were PCR-amplified to about 20 μg/μl. Primers used for PCR amplification were designed for amplicon sequencing according to the instructions of 454 Life Sciences (Branford, CT). The PCR-amplified cDNAs were size-selected using electroelution to obtain products of 119-134 bp. These cDNAs were then sequenced by 454 Life Sciences. The total small RNA reads are 55, 000, 33, 000 and 42, 000 in embryos, sugar-fed midguts (Gut_SF), and blood-fed midguts (Gut_BF), respectively.

### Identification of pre-miRNA sequences in *Ae. aegypti*

Sequences that match known mosquito and *D. melanogaster *pre-miRNAs (miRBase v.12.0, September 2008) were used to identify miRNAs in *Ae. aegypti*. Potential homologues were identified in the genome assemblies of *Ae. aegypti*, *An. gambiae *and *Cx. quinquefasciatus*. The homologous sequences plus 200 bp flanking sequences were retrieved from three mosquito genomes and aligned using Clustalx [38] with a gap open penalty of five and a gap extension penalty of 0.05. The alignments were manually inspected and pre-miRNAs were identified and confirmed by RNAfold http://rna.tbi.univie.ac.at/cgi-bin/RNAfold.cgi using default settings. Eighty-nine pre-miRNAs were identified in *Ae. aegypti *including pre-miRNAs for five previously reported miRNAs that have only been found in mosquitoes so far. Expanding this homologous search approach by using all miRNA and miRNA* sequences in miRBase (September 2008 version) as queries did not yield additional pre-miRNAs in mosquitoes.

To identify novel miRNAs (or pre-miRNAs), we searched the *Ae. aegypti *454 small RNA sequence libraries for sequences that matched both *Ae. aegypti *and *Cx. quinquefasciatus *genome assemblies by BLAST (Ref [39]; e-value cut-off is 0.01). We focused on the comparison between *Ae. aegypti *and *Cx. quinquefasciatus *because these two species are more closely related to each other than to *An. gambiae*. The basic approach is to use sequences in the *Ae. aegypti *small RNA libraries that showed conservation to *Cx. quinquefasciatus *non-coding sequences as leads to find novel miRNAs. Briefly, we first filtered the 454 small RNA sequence libraries by removing sequences that match previously characterized miRNAs, *Ae. aegypti *transposable elements (TEfam: http://tefam.biochem.vt.edu), and other known mosquito non-coding RNAs. We also masked all cDNA transcripts from the *Cx. quinquefasciatus *genome assembly using RepeatMasker http://www.repeatmasker.org/ on a Linux server. We then identified sequences in the masked *Cx. quinquefasciatus *genome that matched the filtered *Ae. aegypti *small RNA libraries using BLASTN (e-value cut-off is 0.01). We then retrieved these matched *Cx. quinquefasciatus *sequences with their flanking genomic sequence as well as the homologous sequences in the *Ae. aegypti *assembly. The homologous sequences from the two species were aligned and folded as described in the above paragraph. Nine novel pre-miRNAs that could produce 7 distinct miRNAs were identified in *Ae. aegypti *during this study (Table 1). Efforts to identify novel miRNAs simply by whole-genome comparison between *Ae. aegypti *and *Cx. quinquefasciatus *did not yield additional miRNAs, nor did efforts based on comparison of 454 small RNA sequences to *Ae. aegypti *genome alone.

To determine whether the novel miRNAs discovered in this study are indeed novel, we used BLAST [39] under low stringent conditions as well as oligomap [31], a program designed for comparisons of short sequences such as miRNAs, allowing gaps and mismatches. BLAST searches were done using low stringent parameters (word size at seven, e-value cut-off at 10) against miRBase and non-redundant GenBank sequences. Oligomap [31] was performed against all miRBase sequences using default parameters.

### 454 sequence count

To determine the number of miRNA and miRNA* hits per sample, 98 *Ae. aegypti *pre-miRNAs identified above were used as query for BLAST analysis. We require 100% match in at least 18 bp for a sequence to be counted. This approach does not distinguish between paralogous pre-miRNAs that produce the same miRNAs, nor does it distinguish between miRNAs that share at least 18 bp perfect match.

### Sample collection for northern blots

For *Ae. aegypti *midgut samples, the sample collection was done the same way as described for preparing samples for 454 sequencing. Sample collections from different developmental stages of *Ae. aegypti*, *An. stephensi*, and *An. gambiae *are briefly described below. Embryo collections were made at 0-12, 12-24, and 24-36 hours after placing a damp collection cup within a cage. To generate points after 12 hours, egg containers were set aside and allowed to incubate at 27°C in a damp collection cup. 0-36 hour samples represent equal mixed pools of 0-12, 12-24 and 24-36 hour samples. Larval samples were collected at each instar, and pooled to generate early (I and II instars) and late (III and IV instars) larvae as listed in each figure. Pupal samples were collected from a pool of varied ages. Adults one to five days following eclosion were collected. In some cases, we did not separate early and late larval samples and used one mixed larval sample instead. These variations are specified in the figure legends of the northern blots. For *T. amboinensis*, embryos were collected at 0-24 hours post-oviposition. Samples were also collected for 1^st ^and 2^nd ^instar larvae and pooled to generate an early larvae sample, and a separate collection of 4^th ^instar larvae was collected for late larvae. Pupae were collected from a pool of varied ages, and male and female adults were collected at two to five days post emergence.

### Northern blot

All samples were either directly processed for RNA isolation or flash frozen on liquid nitrogen immediately following collection, then stored at -80°C. Total RNA isolation was carried out using a mirVana miRNA isolation kit (Ambion, Austin, TX). The amount of total RNA used for each sample is specified in the relevant figure legend. Northern blots were carried out based upon [27]. Briefly, total RNA were loaded onto 15% denaturing polyacrylamide gels, and run beside 19 and 23 nucleotide long ssDNA markers. The RNA gels were transferred to BrightStar-Plus nylon membranes (Ambion), crosslinked using a UV crosslinker, and prehybridized, then hybridized overnight in the ULTRAhyb-Oligo Hybridization Buffer (Ambion) with the appropriate DIG-labeled probe at 42°C. Wash conditions were the same as described in [27]. Antisense 5' digoxigenin-labeled miRCURY LNA probes were purchased from Exiqon (Vedbaek, Denmark). Probe sequences were reverse-complementary to sequences shown in Table 1. The probe for miR-1174 is derived from aga-miR-1174 (miRBase) and it had one base difference to aae-miR-1174.

## Authors' contributions

SLi prepared samples for 454 sequencing, conducted the first northern blot using DIG-labeled probes, performed bioinformatics analysis, provided relevant tables, and wrote a draft of part of the methods section. EAM performed northern analysis on all mosquito-specific miRNAs, worked on the initial bioinformatics analysis on the miR-N1, N2, and N3 cluster, provided relevant figures, assisted in sample dissections, wrote a draft of parts of this manuscript and assisted in revisions. SLiang performed northern analysis on all conserved miRNAs, performed comparisons of miRNA expression in midgut samples, and provided relevant figures. ZT designed and oversaw the project, performed bioinformatics analysis, and wrote most of the manuscript. All authors read and approved the final manuscript.

## Supplementary Material

Additional file 1 **Supplementary table S1**. Supplementary table S1 contains information provided in Table 1 as well as the entire hairpin sequence of each pre-miRNA. Click here for file
